# An Invariant-Based Constitutive Model for Composite Laminates

**DOI:** 10.3390/ma19020409

**Published:** 2026-01-20

**Authors:** Weixian Liu, Shuaijie Fan, Xuefeng Mu, Rufei Ma, Xinfeng Wang

**Affiliations:** 1 AVIC Chengdu Aircraft Design & Research Institute, Chengdu 610041, China; fansj010@avic.com (S.F.);; 2 State Key Laboratory of Mechanics and Control for Aerospace Structures, Nanjing University of Aeronautics and Astronautics, Nanjing 210016, China; xinfengw@nuaa.edu.cn

**Keywords:** composite material mechanics, stiffness invariant theory, the constitutive model of laminate, balanced symmetric layup, reconstruction and simplification

## Abstract

Composite laminates possess complex anisotropic behavior, motivating the development of simplified yet accurate modeling approaches. In this paper, we present a study that introduces a stiffness-invariants-based constitutive model for symmetric, balanced composite laminates, highlighting a novel “quasi-Poisson’s ratio” parameter as a key innovation. The proposed method reconstructs the laminate stiffness matrices using invariant theory (trace of stiffness tensor) and a Master Ply concept, thereby reducing the number of independent material constants. The methods and assumptions (e.g., neglecting minor bending-twisting couplings) are outlined, and the model’s predictions of critical buckling loads are compared to classical laminate theory (CLT) results. Good agreement is observed in most cases, with a consistent conservative bias of CLT. The results confirm that the invariant-based model captures the dominant stiffness characteristics of the laminates and can slightly overestimate stability margins due to its idealizations. In conclusion, this work provides an efficient constitutive modeling framework that can be integrated with finite element analysis and extended to more general laminates in future studies.

## 1. Introduction

With the emergence and widespread application of fiber-reinforced polymer composites in aerospace, automotive, and civil structures, research on the constitutive theories of composite laminates has rapidly gained attention. Advanced composites can be tailored through various architectures—from conventional unidirectional laminates and 2D woven fabrics to through-thickness reinforced laminates (e.g., 3D weaving, stitching, Z-pinning, tufting)—to improve damage tolerance and structural performance [1]. These architectural variations pose challenges for classical analysis methods, as the effective stiffness and stability of a laminate can depend on both the material properties and the stacking sequence/architecture employed. In industrial applications, symmetric and balanced laminate lay-ups are commonly preferred, as they avoid coupling effects and provide quasi-isotropic in-plane behavior, which is advantageous when load directions are uncertain or multi-axial. Such lay-ups ensure that each off-axis ply is paired with an oppositely oriented ply, minimizing anisotropic twist-bend coupling and yielding predictable engineering properties. This practical design preference motivates our focus on symmetric balanced laminates in the present study [2,3].

The Classical Laminate Theory (CLT) [4], the earliest established, simplest in form, and still the most commonly used constitutive model for composite laminates in engineering design, is based on the Kirchhoff hypothesis, which assumes infinite transverse shear stiffness and neglects transverse shear deformation. Consequently, it is only suitable for analyzing and designing thin plate structures. However, composite materials generally exhibit relatively low shear moduli, and the transverse shear stiffness of laminates is much lower than their in-plane stiffness, rendering shear effects often non-negligible. To more accurately capture the mechanical response of thick laminate plates, the First-Order Shear Deformation Theory (FSDT) was developed based on the Reissner–Mindlin plate theory [5,6]. FSDT relaxes the normality assumption, allowing the transverse normal to rotate relative to the mid-surface and thereby accounting for transverse shear deformation. Since the transverse shear strain and stress are assumed constant through the thickness, a deviation from their actual distributions, a shear correction factor is introduced to adjust the constitutive relations and improve computational accuracy. Due to its effectiveness in predicting the global structural response of laminates, FSDT has been widely implemented in many commercial finite element software packages. To more precisely represent the nonlinear shear deformation behaviour of laminates through the thickness, more advanced theoretical models have subsequently been developed, including the High-Order Shear Deformation Theory (HSDT) [7,8], Zig-Zag Theory [9,10], and Layer-Wise Theory [11,12]. These models more faithfully reproduce the thickness-wise distribution of shear strain and significantly improve the predictive capability for thick laminates. Nonetheless, their increased theoretical complexity has limited widespread adoption in practical composite laminate analysis and design.

The mechanical analysis and design of composite materials are considerably more complex than those of isotropic materials, owing to their inherent anisotropy and multilayered architecture. This complexity typically requires extensive material parameters as inputs and relies on classical laminate theory to determine the constitutive stiffness matrix. As a result, any changes in material selection, ply percentage, or stacking sequence often requires repeated mechanical analyses and experimental validation, leading to prolonged duration and high costs. These challenges have motivated continued efforts in the academic community to develop more efficient and fundamental analysis and design approaches. In this context, the theory of stiffness invariants has been progressively established. This theory stems from a crucial insight, i.e., certain combination of its components known as invariants remain unchanged regardless of coordinate selection, although the stiffness matrix of composite materials varies with coordinate system rotation. These invariants capture the intrinsic stiffness characteristics of the material. As early as 1968, Tsai and Pagano [13] introduced a transformation that transformed the power-function representation of the off-axis stiffness into a “U-invariant” expression composed of four independent constants and double-angle trigonometric terms. Through combination of these U-invariants, they further revealed the trace invariant of the stiffness matrix, denoted in this paper as the “L-invariant”. In 2014, Tsai demonstrated that the normalized stiffness trace Tr(Q) of various carbon-fibre composites could be treated as a new independent material constant through extensive statistical analysis [14,15]. By 2020, this quantity was formally named the “Tsai’s modulus”. With further research, the concept of the “Master Ply” was subsequently proposed, greatly simplifying the characterization and preliminary design process for composite materials and structures [16,17,18]. Indeed, the “Master Ply” concept introduced by Tsai & Melo takes advantage of this by defining a representative ply with median normalized properties that can serve as a baseline for any laminate made of similar plies. In other words, across a broad sample of carbon/epoxy laminas, key normalized stiffness ratios were found to cluster tightly, allowing one set of averaged properties⁠—a Master Ply—to represent the whole family [19].

Parallel to these theoretical advances, researchers are increasingly interested in linking such invariant-based models with actual structural behavior and performance. For example, recent work has applied invariant concepts to laminate optimization in aerospace structures, demonstrating simpler yet effective design processes [20]. At the same time, comprehensive reviews have highlighted the need for more efficient multiscale modeling and reduced-order approaches for composite damage and failure prediction [21]. These trends underscore the significance of developing constitutive models that balance simplicity with accuracy—providing engineers with deeper design insight and computational efficiency, without sacrificing predictive capability. Our work contributes to this direction by reconstructing the constitutive model of a composite laminate using stiffness invariants theory, and by introducing a new parameter termed the “quasi-Poisson’s ratio” to capture out-of-plane deformation effects. We aim to clarify the physical meaning of this quasi-Poisson’s ratio and demonstrate its influence on laminate behavior, thereby connecting the invariant-based theoretical framework to tangible engineering implications (e.g., buckling stability and local deformation characteristics).

In summary, the objective of this paper is to develop an invariant-based constitutive modeling method for symmetric composite laminates and to evaluate its performance against classical theory. The novelty lies in: (a) the reconstruction of laminate matrix A and matrix D using a Master Ply approach (drastically reducing required input parameters), and (b) the definition of a “quasi-Poisson’s ratio” for laminates, which provides a simplified measure of the laminate’s effective Poisson effect in bending. The following sections describe the methodology, including materials selection and assumptions, then present the model derivations and a discussion of results such as critical buckling load predictions. The practical significance of this method is also discussed—in particular, how it can improve computational efficiency in design (by using just a few invariant parameters) and enhance understanding of laminate anisotropy effects on structural performance.

## 2. Materials and Methods

### 2.1. Materials

To justify our modeling approach, we first consider the material widly used in industry. Table 1 summarizes typical composite material property data cited from reference [22]. The data comprise various reinforcing fibers (glass, aramid, carbon, and boron fibers), matrices (thermoset epoxy, thermoplastic PEEK, and aluminum), and fiber architectures (unidirectional and fabric).

### 2.2. Stiffness Invariant of the Lamina

The “U-invariants” for the off-axis stiffness ${\bar{Q}}_{ij}$ of the lamina were first established and derived in detail by Tsai and Pagano [13]. This formulation has since been widely incorporated into textbooks and handbooks on composite mechanics and structural design, and is given by the following expression:

(1) $$\begin{array}{l} {\bar{Q}}_{11}=U_{1}+U_{2}\cos2\theta +U_{3}\cos4\theta \\ {\bar{Q}}_{22}=U_{1}-U_{2}\cos2\theta +U_{3}\cos4\theta \\ {\bar{Q}}_{12}=U_{4}-U_{3}\cos4\theta \\ {\bar{Q}}_{66}=U_{5}-U_{3}\cos4\theta \end{array}$$

where coupling terms are omitted, θ denotes the off-axis angle and the U-invariant is defined as follows:

(2) $$\begin{array}{l} U_{1}=\frac{1}{8}\left(3Q_{11}+3Q_{22}+2Q_{12}+4Q_{66}\right)\\ U_{2}=\frac{1}{2}\left(Q_{11}-Q_{22}\right)\\ U_{3}=\frac{1}{8}\left(Q_{11}+Q_{22}-2Q_{12}-4Q_{66}\right)\\ U_{4}=\frac{1}{8}\left(Q_{11}+Q_{22}+6Q_{12}-4Q_{66}\right)\\ U_{5}=\frac{1}{2}\left(U_{1}-U_{4}\right)=\frac{1}{8}\left(Q_{11}+Q_{22}-2Q_{12}-4Q_{66}\right) \end{array}$$

In accordance with the mechanics of composite materials [4,22], the components of the on-axis stiffness $Q_{ij}$ for lamina are expressed using engineering constants as follows:

(3) $$\begin{array}{cccc} Q_{11}=\frac{E_{1}}{1-\nu_{12}\nu_{21}} & Q_{22}=\frac{E_{2}}{1-\nu_{12}\nu_{21}} & Q_{12}=\frac{\nu_{21}E_{1}}{1-\nu_{12}\nu_{21}}=\frac{\nu_{12}E_{2}}{1-\nu_{12}\nu_{21}} & Q_{66}=G_{12} \end{array}$$

“L invariant” for the stiffness of lamina was introduced in the same reference [13] simultaneously. This quantity was subsequently established as the “trace invariant” [14], denoted as Tr(**Q**) and now recognized as “Tsai modulus”, as given by the following equation:

(4) $$\begin{array}{l} L_{1}=Tr\left(Q\right)=Q_{11}+Q_{22}+2Q_{66}=2\left(U_{1}-U_{5}\right)\\ L_{2}=Q_{66}-Q_{12}=U_{5}-U_{4} \end{array}$$

Substituting Equation (3) into Equation (4) yields:

(5) $$\begin{array}{cc} L_{1}=\frac{E_{1}+E_{2}}{1-\nu_{12}\nu_{21}}+2G_{12} & L_{2}=G_{12}-\frac{\nu_{21}E_{1}}{1-\nu_{12}\nu_{21}} \end{array}$$

For the sake of convenience in subsequent expressions within this paper, the “quasi-Poisson’s ratio” *η* for anisotropic lamina is introduced and defined as the ratio of invariant *L*_2_ to invariant *L*_1_, expressed as:

(6) $$\eta =\frac{L_{2}}{L_{1}}=\frac{Q_{66}-Q_{12}}{Q_{11}+Q_{22}+2Q_{66}}\times 100\%$$

The quasi-Poisson’s ratio *η* can be conceptually understood as analogous to the “Poisson’s ratio” in isotropic materials. Thus, through the stiffness invariants of lamina, the “Tsai modulus” and “quasi-Poisson’s ratio” together enable an analogy between the stiffness characteristics of anisotropic lamina and the Young’s modulus and Poisson’s ratio of isotropic materials.

Substituting Equation (5) into the preceding expression yields the formula for the quasi-Poisson ratio of a lamina:

(7) $$\eta =\frac{\left(1-\nu_{12}\nu_{21}\right)G_{12}-\nu_{21}E_{1}}{2\left(1-\nu_{12}\nu_{21}\right)G_{12}+E_{1}+E_{2}}\times 100\%$$

### 2.3. Master Ply

The term “Tsai’s modulus” was adopted by Tsai [14] for normalizing the stiffness of different carbon fiber composites and its definition is given as follows:

(8) $$Q_{ij}=Q_{ij}^{*}\times Tr\left(Q\right)$$

Through extensive statistical analysis, Tsai found that the stiffness properties of various laminae exhibited minimal variation after normalization. Adopting the statistical mean of these values established the concept of the “Master Ply”, which represented generalized material properties set on a statistical basis. Table 1 compiles Tsai’s statistical results for the “Master Ply” together with the data from Jia [23] for 228 materials, and includes the corresponding calculated quasi-Poisson ratio *η*.

In Table 2, the “L invariant” and quasi-Poisson’s ratio *η* are also included. The quasi-Poisson’s ratio has a mean value of 1.49% and a maximum value of 2.55%.

The primary observation from Table 1 and Table 2 is that the quasi-Poisson’s ratio *η* of the Master Ply is small (1.78%), signifying that the contribution of invariant L_2_ to the total invariant L_1_ is relatively low.

### 2.4. Stiffness Invariant of the Laminate

According to the classical laminate theory of composite materials [4,22], the constitutive relationship between the internal force **N** and internal moment **M** per unit width and the mean-plane strain **ε**^0^ and curvature **κ** is as follows:

(9) $$\left[\begin{array}{c} N\\ M \end{array}\right]=\left[\begin{array}{cc} A & B\\ B & D \end{array}\right]\left[\begin{array}{c} \varepsilon^{0}\\ \kappa \end{array}\right]$$

For the symmetrical balanced laminate composed of the same lamina studied in this paper, the extension-bending coupling stiffness matrix **B** vanishes, likewise the extension-shear coupling terms *A*_16_ and *A*_26_ are zero. Consequently, the equation is simplified to:

(10) $$\begin{array}{c} \left[\begin{array}{c} N_{x}\\ N_{y}\\ N_{xy} \end{array}\right]=\left[\begin{array}{ccc} A_{11} & A_{12} & 0\\ A_{12} & A_{22} & 0\\ 0 & 0 & A_{66} \end{array}\right]\left[\begin{array}{c} \varepsilon_{x}^{0}\\ \varepsilon_{y}^{0}\\ \gamma_{xy}^{0} \end{array}\right]\\ \left[\begin{array}{c} M_{x}\\ M_{y}\\ M_{xy} \end{array}\right]=\left[\begin{array}{ccc} D_{11} & D_{12} & D_{16}\\ D_{12} & D_{22} & D_{26}\\ D_{16} & D_{26} & D_{66} \end{array}\right]\left[\begin{array}{c} \kappa_{x}\\ \kappa_{y}\\ \kappa_{xy} \end{array}\right] \end{array}$$

The component of the in-plane extensional stiffness matrix **A** and the out-of-plane bending stiffness matrix **D** are defined as follows:

(11) $$\begin{array}{cc} A_{ij}=\sum_{k=1}^{n}{\left({\bar{Q}}_{ij}\right)}_{k}\left(z_{k}-z_{k-1}\right) & D_{ij}=\frac{1}{3}\sum_{k=1}^{n}{\left({\bar{Q}}_{ij}\right)}_{k}\left(z_{k}^{3}-z_{k-1}^{3}\right) \end{array}$$

Based on Equations (4) and (11), Tsai [12] established the invariants for both the in-plane extensional stiffness matrix **A** and the out-of-plane bending stiffness matrix **D**, as given by:

(12) $$\begin{array}{l} A_{1}=A_{11}+A_{22}+2A_{66}=t\left(Q_{11}+Q_{22}+2Q_{66}\right)=L_{1}t\\ A_{2}=A_{66}-A_{12}=t\left(Q_{66}-Q_{12}\right)=L_{2}t\\ D_{1}=D_{11}+D_{22}+2D_{66}=\frac{t^{3}}{12}\left(Q_{11}+Q_{22}+2Q_{66}\right)=\frac{L_{1}t^{3}}{12}\\ D_{2}=D_{66}-D_{12}=\frac{t^{3}}{12}\left(Q_{66}-Q_{12}\right)=\frac{L_{2}t^{3}}{12} \end{array}$$

where *t* denotes the total thickness of the laminate.

Substituting Equation (12) into the definition of the quasi-Poisson’s ratio *η* given by Equation (6) yields:

(13) $$\eta =\frac{L_{2}}{L_{1}}=\frac{A_{2}}{A_{1}}=\frac{D_{2}}{D_{1}}$$

This indicates that the laminate exhibits the same quasi-Poisson’s ratio as its constituent lamina.

## 3. Research on the Constitutive Model of Laminate

### 3.1. Stiffness Ratio Definition and Constitutive Reconstruction

A new parameter termed the “design stiffness ratio” is introduced to characterize the layup. The introduction of “design stiffness ratio” can decouple the material properties, captured by the stiffness invariants of the lamina, from the layup design variables (ply percentage and stacking sequence), thereby simplify the stiffness matrix calculation for symmetric balanced laminates. The “design stiffness ratio” is defined by:

(14) $$\begin{array}{cccc} \xi_{A}=\frac{A_{66}}{A_{1}} & \zeta_{A}=\frac{A_{11}}{A_{11}+A_{22}} & \xi_{D}=\frac{D_{66}}{D_{1}} & \zeta_{D}=\frac{D_{11}}{D_{11}+D_{22}} \end{array}$$

Here, *ξ_A_* denotes the ratio of in-plane shear stiffness to the invariant A_1_ of the in-plane stiffness matrix; *ζ_A_* denotes the proportion of the tensile stiffness in x-direction relative to the total tensile stiffness in x- and y-directions; *ξ_D_* denotes the ratio of out-of-plane torsional stiffness to the invariant D_1_ of the bending stiffness matrix; *ζ_D_* denotes the proportion of the bending stiffness in x-direction relative to the total bending stiffness in the x- and y-directions.

Substituting Equation (14) into Equations (11) and (12) and rearranging obtain the individual components of the stiffness matrix as follows:

(15) $$\begin{array}{l} A_{11}=\zeta_{A}\left(1-2\xi_{A}\right)A_{1}=\zeta_{A}\left(1-2\xi_{A}\right)L_{1}t\\ A_{22}=\left(1-\zeta_{A}\right)\left(1-2\xi_{A}\right)A_{1}=\left(1-\zeta_{A}\right)\left(1-2\xi_{A}\right)L_{1}t\\ A_{12}=\xi_{A}A_{1}-A_{2}=\left(\xi_{A}-\eta \right)L_{1}t\\ A_{66}=\xi_{A}A_{1}=\xi_{A}L_{1}t\\ D_{11}=\zeta_{D}\left(1-2\xi_{D}\right)D_{1}=\zeta_{D}\left(1-2\xi_{D}\right)\frac{L_{1}t^{3}}{12}\\ D_{22}=\left(1-\zeta_{D}\right)\left(1-2\xi_{D}\right)D_{1}=\left(1-\zeta_{D}\right)\left(1-2\xi_{D}\right)\frac{L_{1}t^{3}}{12}\\ D_{12}=\xi_{D}D_{1}-D_{2}=\left(\xi_{D}-\eta \right)\frac{L_{1}t^{3}}{12}\\ D_{66}=\xi_{D}D_{1}=\xi_{D}\frac{L_{1}t^{3}}{12} \end{array}$$

In conventional design of engineering composite structures, the layup sequence is typically configured to minimize the bending-torsion coupling, i.e., the terms *D*_16_ and *D*_26_. Consequently, bending-torsion coupling is neglected in this work by setting *D*_16_ = *D*_26_ = 0. Rearranging Equation (15) into matrix form and explicitly separating terms related to the stiffness invariant *L*_1_ (also termed the ‘Tsai modulus’) from those associated with the total thickness *t* yields the reconstructed in-plane extensional stiffness matrix **A** and out-of-plane bending stiffness matrix **D** as follows:

(16) $$\begin{array}{l} A=\left[\begin{array}{ccc} \zeta_{A}\left(1-2\xi_{A}\right) & \xi_{A}-\eta & 0\\ \xi_{A}-\eta & \left(1-\zeta_{A}\right)\left(1-2\xi_{A}\right) & 0\\ 0 & 0 & \xi_{A} \end{array}\right]L_{1}t\\ D=\left[\begin{array}{ccc} \zeta_{D}\left(1-2\xi_{D}\right) & \xi_{D}-\eta & 0\\ \xi_{D}-\eta & \left(1-\zeta_{D}\right)\left(1-2\xi_{D}\right) & 0\\ 0 & 0 & \xi_{D} \end{array}\right]L_{1}\frac{t^{3}}{12} \end{array}$$

### 3.2. Constitutive Matrix Simplification

Based on Tsai’s statistical analysis of the ‘Master Ply’ for carbon-fibre-reinforced polymer composites [14,15], the mean value of the quasi-Poisson’s ratio *η* is 1.78%, as given in Table 1. Substituting this value into Equation (16) yields the in-plane extensional stiffness matrix **A** and out-of-plane bending stiffness matrix **D** for the symmetrical balanced laminate of the ‘Master Ply’ as follows:

(17) $$\begin{array}{l} A_{Master}=\left[\begin{array}{ccc} \zeta_{A}\left(1-2\xi_{A}\right) & \xi_{A}-0.0178 & 0\\ \xi_{A}-0.0178 & \left(1-\zeta_{A}\right)\left(1-2\xi_{A}\right) & 0\\ 0 & 0 & \xi_{A} \end{array}\right]L_{1}t\\ D_{Master}=\left[\begin{array}{ccc} \zeta_{D}\left(1-2\xi_{D}\right) & \xi_{D}-0.0178 & 0\\ \xi_{D}-0.0178 & \left(1-\zeta_{D}\right)\left(1-2\xi_{D}\right) & 0\\ 0 & 0 & \xi_{D} \end{array}\right]L_{1}\frac{t^{3}}{12} \end{array}$$

Owing to the characteristically low shear modulus of composite materials, the quasi-Poisson’s ratio η, as indicated by Equation (7) and Table 1 and Table 2, is notably small. Given its small magnitude, η is assumed to be zero to facilitate further simplification, implying *Q*_66_ = *Q*_12_. Under this assumption, the in-plane extensional stiffness matrix **A** and out-of-plane bending stiffness matrix **D** for a symmetrical balanced laminate can be approximated in the following form:

(18) $$\begin{array}{l} A\approx \tilde{A}=\left[\begin{array}{ccc} \zeta_{A}\left(1-2\xi_{A}\right) & \xi_{A} & 0\\ \xi_{A} & \left(1-\zeta_{A}\right)\left(1-2\xi_{A}\right) & 0\\ 0 & 0 & \xi_{A} \end{array}\right]L_{1}t\\ D\approx \tilde{D}=\left[\begin{array}{ccc} \zeta_{D}\left(1-2\xi_{D}\right) & \xi_{D} & 0\\ \xi_{D} & \left(1-\zeta_{D}\right)\left(1-2\xi_{D}\right) & 0\\ 0 & 0 & \xi_{D} \end{array}\right]L_{1}\frac{t^{3}}{12} \end{array}$$

where the overbar on a matrix denotes the approximation of the corresponding matrix.

### 3.3. Boundary Values of the Stiffness Ratio

According to the mechanics of composite materials [4,22], the maximum value of ${\bar{Q}}_{11}$ for lamina occurs at an off-axis angle of 0° and the minimum one occures at an off-axis angle of 90°. Just opposite, the maximum value of ${\bar{Q}}_{22}$ appears at an off-axis angle of 90° and the minimum one occurs at an off-axis angle of 0°. Simultaneously, the ${\bar{Q}}_{66}$ reaches its maximum at ±45°, whilst attaining its minimum at 0° or 90°.

Accordingly, combining the definition of the design stiffness ratio by Equation (15) reveals that when the laminate consists entirely of ±45° layers, the stiffness ratios *ξ_A_* and *ξ_D_* attain their maximum values, whilst *ζ_A_* and *ζ_D_* assume the intermediate value of 0.5 due to identical off-axis stiffness in the x and y directions. When the laminate consists entirely of 0° or 90° layers, *ξ_A_* and *ξ_D_* attain their minimum values. However, a distinction is required for *ζ_A_* and *ζ_D_*. When the layup is [0]_n_, *ζ_A_* and *ζ_D_* reach their maximum values; when the layup is [90]_n_, *ζ_A_* and *ζ_D_* attain their minimum values.

The boundary extreme values discussed above are compiled in Table 3 to facilitate the design and optimization of composite laminates.

## 4. Validation and Application

To validate the effectiveness of the proposed constitutive model for symmetrical balanced laminate design, the maximum quasi-Poisson’s ratio η among unidirectional composites is 2.44% as listed in Table 2. Consequently, the case study selected the corresponding AS4/3501-6 carbon-fibre-reinforced epoxy composite material, with a Young’s modulus of 172.11 GPa and a single-layer thickness of 0.125 mm, and the remaining material properties are detailed in Table 2.

A rectangular laminated plate measuring 200 mm × 600 mm is selected for case study and validations. Numerical simulations were conducted using the commercial finite element software ABAQUS. Multiple typical loading conditions were applied. Ten common laminate configurations listed in Table 4 were examed, encompassing key design variables such as total thickness, laminate ratio, and lay-up sequence. Table 4 also presents the design stiffness ratios for each laminate group, calculated using Equation (14), for subsequent analysis and comparison.

### 4.1. Geometric Nonlinear Analysis Under Out-of-Plane Uniform Pressure

**Figure 1:**
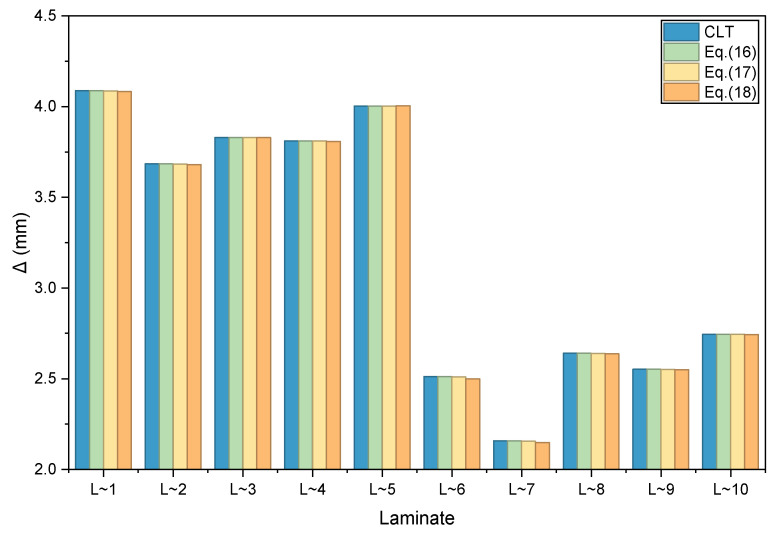
Comparisons of geometrically nonlinear analysis results under out-of-plane uniform pressure.

The verification case considers a rectangular laminated plate simply supported on all four sides. A geometrically nonlinear analysis is performed under an out-of-plane uniformly distributed pressure of 0.1 MPa, accounting for the coupling between membrane and bending effects due to deformation. All translational degrees of freedom on four edges are constrained. Numerical calculations are carried out for the 10 typical layup designs listed in Table 4. The corresponding maximum displacement Δ for each design case is summarized in Table 5 and Figure 1. Additionally, Table 5 presents the deviation of the results obtained from the reconstructed and simplified constitutive model proposed in this study relative to those from the Classical Lamination Theory (CLT).

As evidenced by the data listed in Table 5 and the bar chart shown in Figure 1, two conclusions may be drawn. (1) The analysis results obtained using the stiffness matrices derived from the nominal lamination parameters are consistent with those from the Classical Lamination Theory (CLT). This indicates that when the bending-twisting coupling terms (D_16_, D_26_) are small in practical laminate sequence designs, neglecting these terms has no noticeable effect on the out-of-plane deformation under uniform pressure. (2) The deviations in the results based on the primary laminate stiffness matrices and the simplified approximate stiffness matrices are minimal. The maximum error observed for the primary stiffness matrices is only 0.119%, while that for the simplified approximate stiffness matrices is 0.518%. Both error levels are considered negligible in engineering practice.

### 4.2. Linear Buckling Analysis Under In-Plane Uniaxial Compression

**Figure 2:**
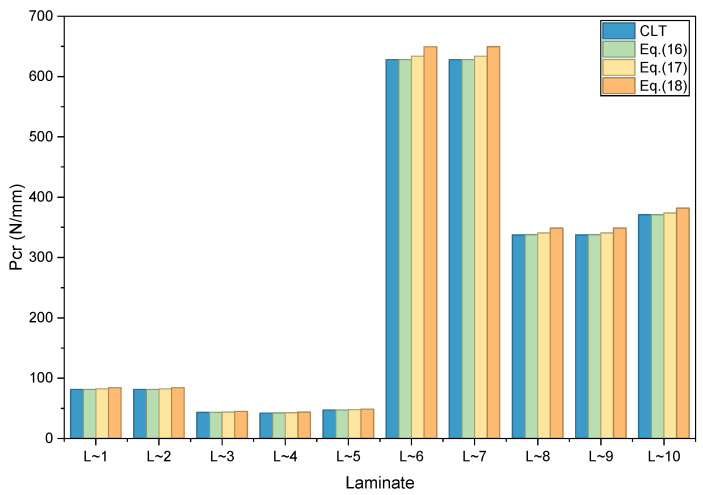
Comparisons of linear buckling analysis results under in-plane axial compression.

In this verification case, a simply supported rectangular laminated plate subjected to a uniformly distributed in-plane compressive load applied along the short edge (parallel to the longitudinal axis) is considered. Linear buckling analyses are performed for the ten typical lay-up designs. The corresponding critical buckling loads (Pcr) are summarized in Table 6 and illustrated in Figure 2. Additionally, Table 6 also provides the deviations between the results obtained from the reconstructed and simplified constitutive models proposed in this work and those derived from the Classical Lamination Theory (CLT).

Based on the data in Table 6 and the bar chart in Figure 2, following observations can be made when comparing the results with those from the Classical Lamination Theory (CLT):

(1) As the stiffness matrix of the laminate is reconstructed and progressively simplified, the predicted critical compressive buckling load shows a sequential increase, accompanied by a growing analysis error.
(2) The maximum error in the critical buckling load obtained using the designed stiffness ratio (reconstructed stiffness matrix) is 0.477%. For the analysis based on the principal layup stiffness matrix, the maximum error reaches 1.368%, whereas the result derived from the simplified approximate stiffness matrix exhibits a maximum error of 3.759%.
(3) The designed stiffness ratio (reconstructed stiffness matrix) only neglects the bending–twisting coupling terms, which have a minor influence on the in-plane uniaxial compressive buckling behavior. Although the simplified approximate stiffness matrix introduces certain errors, its accuracy remains acceptable in the engineering design stage, demonstrating good practical utility.

### 4.3. Linear Buckling Analysis Under In-Plane Shear

**Figure 3:**
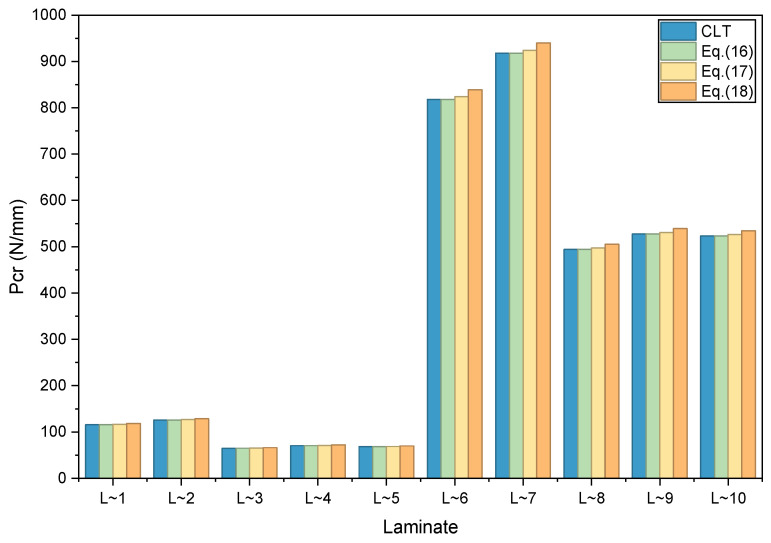
Comparisons of Linear Buckling Analysis Results under In-Plane Shear.

In the verification case of in-plane shear buckling, a simply supported rectangular laminated plate subjected to uniformly distributed in-plane shear loads is considered. Linear buckling analyses are performed for the ten typical lay-up designs. The corresponding critical buckling loads (Pcr) for each design case, together with their deviations from the results obtained via Classical Lamination Theory (CLT), are summarized in Table 7 and illustrated in Figure 3.

It should be noted that due to the presence of the bending-torsion coupling effect, the results predicted by classical laminated theory manifest as two critical load values: the positive shear critical buckling load (denoted by “+” in the table) and the negative shear critical buckling load (denoted by “−” in the table). For the sake of comparison, the average of the absolute values of these two loads is taken as the reference value P_cr_ for CLT, and the error is calculated based on this.

As indicated by the data in Table 7 and the comparative bar chart in Figure 3, following conclusions can be drawn relative to the results obtained from the Classical Lamination Theory (CLT):

(1) Consistent with the aforementioned uniaxial compression buckling case, as the degree of simplification in the stiffness matrix increases, the predicted shear buckling load gradually rises, accompanied by a stepwise increase in the analytical error.
(2) The maximum error in the critical buckling load obtained using the designed stiffness ratio (reconstructed stiffness matrix) is 0.088%. For the results based on the principal layup stiffness matrix, the maximum error reaches 0.739%, whereas the result derived from the simplified approximate stiffness matrix exhibits a maximum error of 2.571%.
(3) Although the designed stiffness ratio (reconstructed stiffness matrix) neglects the bending–twisting coupling terms, its influence on the in-plane shear buckling response remains limited. This systematic overestimation aligns with our expectations and is now explicitly discussed: because our model uses averaged stiffness values (which do not account for certain compliance effects of specific stacking sequences), it effectively assumes a somewhat “stiffer” laminate than some actual lay-ups, thus yielding higher (non-conservative) buckling loads. Despite introducing relatively larger errors, the simplified approximate stiffness matrix still yields accuracy within an acceptable range for the engineering design stage, demonstrating certain practical and reference value.

## 5. Conclusions

Based on the theory of stiffness invariants, this paper investigates symmetrically balanced composite laminates composed of identical single-layer materials. By defining the quasi-Poisson’s ratio of the single-layer material and the design stiffness ratio of the laminate, reconstruction and simplification of the in-plane stiffness and out-of-plane bending stiffness matrices within classical laminate theory can be achieved. This provides a more convenient approach for laminate design and optimization. The principal conclusions are as follows:

(1) The quasi-Poisson ratio defined based on the stiffness invariant, together with the Young’s modulus, jointly characterizes the mechanical properties of anisotropic laminated materials. Its role in composite materials is analogous to that of Young’s modulus and Poisson’s ratio in isotropic materials.
(2) The reconfigured and simplified constitutive stiffness matrix within the classical laminate theory achieves separation between single-layer material parameters (Cai’s modulus and quasi-Poisson’s ratio) and laminate layup characteristics (design stiffness ratio), offering novel approaches for material design and layup optimization in laminates.
(3) Verification through numerical examples of common layering designs under various typical operating conditions demonstrates that the reconfigured and simplified constitutive model proposed in this paper exhibits minimal error. Within the permissible range of engineering design, the model possesses sound practicality and effectiveness.

However, the model also has clear limitations. It does not account for unsymmetric lay-ups, extreme anisotropy (e.g., all fibers aligned in one direction), or lay-ups where stacking sequence (not just overall percentages) critically affects the response. The model also currently assumes a single material system (the Master Ply) and thus may lose accuracy if applied outside the material property range used in its calibration (for example, applying it to a composite with significantly higher fiber volume fraction or a different constituent would require recalibrating the invariants). Users of the model should be aware that detailed through-thickness stress distributions are not captured. Thus, for failure predictions like first-ply failure or interlaminar stresses, a more refined analysis would be necessary. Future work will aim to extend the invariant-based approach beyond these limitations. One promising direction is the integration with Finite Element Analysis (FEA): the invariant model could serve as a plugin or user material in FE codes to provide rapid laminate behavior predictions, even under complex boundary conditions, as a “lightweight” alternative to full ply-by-ply modeling. We believe this approach offers both theoretical insight and engineering utility, and we have outlined a path for its expansion to address more complex cases and integration with comprehensive composite design tools.

We also plan to investigate the model’s applicability to dynamic loading and impact scenarios, examining whether a similar reduction in parameters can capture essential response features under transient loads. Incorporating thermo-mechanical effects (e.g., thermal expansion invariants or temperature-dependent stiffness changes) is another extension, which would be important for applications in varying thermal environments. Extending the theory to unsymmetric or unbalanced laminates will require including additional invariants or parameters (for example, to account for coupling matrices **B** or non-zero **D**_16_ and **D**_26_, but could broaden the model’s use significantly.

## Figures and Tables

**Table 1:** Material Properties of Typical Lamina [22].

		*V*_f_	*E*_11_/GPa	*E*_22_/GPa	*ν*_12_	*G*_12_/GPa	*L*_1_/GPa	*L*_2_/GPa	*η*/%
E-Glass/Epoxy	-	0.55	41	10.4	0.28	4.3	61.04	1.33	2.18
Kevlar/Epoxy	Aramid 49/Epoxy	0.66	80	5.5	0.34	2.2	90.58	0.32	0.35
Carbon/Epoxy	AS4/3501-6	0.63	147	10.3	0.27	7	172.11	4.20	2.44
Carbon/Epoxy	IM7/997-3	0.65	190	9.9	0.35	7.8	216.78	4.31	1.99
Carbon/PEEK	AS4/APC2	0.58	138	8.7	0.28	5.0	157.43	2.55	1.62
Graphite/Epoxy	GY-70/934	0.57	294	6.4	0.23	4.9	310.55	3.43	1.10
Boron/Epoxy	B5.6/5505	0.5	201	21.7	0.17	5.4	234.20	1.70	0.73
Boron/Aluminum	B4/6061-Al	0.5	235	137	0.3	47	486.60	3.62	0.74
Woven Glass/Epoxy	120/3501-6	0.55	27.5	26.7	0.14	5.5	66.25	1.69	2.55
Carbon Fabric/Epoxy	AGP370-5H/3501-6S	0.62	77	75	0.06	6.5	165.53	1.98	1.20
Average									1.49
Max									2.55

**Table 2:** Statistical Values of Master Ply.

	$Q_{11}^{*}$	$Q_{22}^{*}$	$Q_{12}^{*}$	$Q_{66}^{*}$	*η*/%
Master Ply [14]	88.15%	4.99%	1.64%	3.42%	1.78%
Jia [23]	88.5%	5.3%	1.6%	2.9%	1.3%

**Table 3:** Boundary Values of the Stiffness Ratio.

	*ξ_A_* and *ξ_D_*		*ζ_A_* and *ζ_D_*	
[±45]_ns_	max	$\frac{Q_{11}+Q_{22}-2Q_{12}}{4\left(Q_{11}+Q_{22}+2Q_{66}\right)}$	-	0.5
[0]_n_	min	$\frac{Q_{66}}{Q_{11}+Q_{22}+2Q_{66}}$	max	$\frac{Q_{11}}{Q_{11}+Q_{22}}$
[90]_n_			min	$\frac{Q_{22}}{Q_{11}+Q_{22}}$

**Table 4:** Typical Laminate Stacking Sequences.

Laminate	Lay-Up	t/mm	Percentage	*ζ_A_*	*ξ_A_*	*ζ_D_*	*ξ_D_*
L~1	[45/90/−45/0/0/−45/0/0/45/0]_s_	2.5	50:40:10	0.706	0.113	0.532	0.133
L~2	[45/90/−45/0/90/−45/0/0/45/0]_s_	2.5	40:40:20	0.603	0.113	0.433	0.133
L~3	[45/90/−45/0/−45/0/45/90]_s_	2.0	25:50:25	0.500	0.131	0.447	0.148
L~4	[45/90/−45/90/−45/0/45/0]_s_	2.0	25:50:25	0.500	0.131	0.314	0.148
L~5	[45/−45/90/−45/45/0/45/−45]_s_	2.0	10:80:10	0.500	0.176	0.412	0.183
L~6	[45/90/−45/0/0/−45/0/0/45/0]_2s_	5.0	50:40:10	0.706	0.113	0.626	0.122
L~7	[45/90/−45/0/90/−45/0/0/45/0]_2s_	5.0	40:40:20	0.603	0.113	0.519	0.122
L~8	[45/90/−45/0/−45/0/45/90]_2s_	4.0	25:50:25	0.500	0.131	0.487	0.140
L~9	[45/90/−45/90/−45/0/45/0]_2s_	4.0	25:50:25	0.500	0.131	0.403	0.140
L~10	[45/−45/90/−45/45/0/45/−45]2s	4.0	10:80:10	0.500	0.176	0.456	0.178

**Table 5:** Results of Geometric Nonlinear Analysis under Out-of-Plane Uniform Pressure.

Laminate	CLT	Equation (16)		Equation (17)		Equation (18)	
	Δ/mm	Δ/mm	Error/%	Δ/mm	Error/%	Δ/mm	Error/%
L~1	4.089	4.089	0.000	4.087	−0.049	4.083	−0.147
L~2	3.684	3.684	0.000	3.683	−0.027	3.681	−0.081
L~3	3.831	3.831	0.000	3.830	−0.026	3.829	−0.052
L~4	3.810	3.810	0.000	3.810	0.000	3.808	−0.052
L~5	4.004	4.004	0.000	4.004	0.000	4.005	0.025
L~6	2.512	2.512	0.000	2.509	−0.119	2.499	−0.518
L~7	2.157	2.157	0.000	2.155	−0.093	2.148	−0.417
L~8	2.640	2.640	0.000	2.639	−0.038	2.637	−0.114
L~9	2.552	2.552	0.000	2.551	−0.039	2.549	−0.118
L~10	2.745	2.745	0.000	2.745	0.000	2.743	−0.073
Max Abs			0.000		0.119		0.518

**Table 6:** Results of Linear Buckling Analysis under Uniaxial In-Plane Compression.

Laminate	CLT	Equation (16)		Equation (17)		Equation (18)	
	P_cr_/(N/mm)	P_cr_/(N/mm)	Error/%	P_cr_/(N/mm)	Error/%	P_cr_/(N/mm)	Error/%
L~1	81.255	81.336	0.100	82.093	1.031	84.059	3.451
L~2	81.264	81.360	0.118	82.093	1.020	84.059	3.439
L~3	43.246	43.435	0.437	43.810	1.304	44.817	3.633
L~4	42.113	42.314	0.477	42.689	1.368	43.696	3.759
L~5	47.215	47.261	0.097	47.636	0.892	48.643	3.024
L~6	628.05	628.06	0.002	633.90	0.931	649.57	3.426
L~7	628.07	628.08	0.002	633.92	0.931	649.60	3.428
L~8	337.71	337.81	0.030	340.80	0.915	348.84	3.296
L~9	337.71	337.80	0.027	340.79	0.912	348.83	3.293
L~10	370.74	370.84	0.027	373.83	0.833	381.87	3.002
Max			0.477		1.368		3.759

**Table 7:** Results of Linear Buckling Analysis under In-Plane Shear.

Laminate	CLT			Equation (16)		Equation (17)		Equation (18)	
	+	−	P_cr_/(N/mm)	P_cr_/(N/mm)	Error/%	P_cr_/(N/mm)	Error/%	P_cr_/(N/mm)	Error/%
L~1	111.33	−119.49	115.41	115.43	0.017	116.17	0.659	118.14	2.365
L~2	121.43	−129.76	125.60	125.62	0.020	126.39	0.633	128.45	2.273
L~3	60.435	−68.708	64.572	64.615	0.067	65.002	0.667	66.035	2.266
L~4	65.710	−74.714	70.212	70.274	0.088	70.731	0.739	71.950	2.475
L~5	66.104	−70.235	68.170	68.180	0.015	68.56	0.573	69.577	2.065
L~6	815.10	−821.12	818.11	818.11	0.000	823.83	0.699	839.14	2.571
L~7	914.95	−921.03	917.99	917.99	0.000	923.92	0.646	939.81	2.377
L~8	485.88	−502.22	494.05	494.07	0.004	497.12	0.621	505.27	2.271
L~9	519.01	−535.69	527.35	527.38	0.006	530.55	0.607	539.04	2.217
L~10	515.33	−531.66	523.50	523.51	0.003	526.5	0.574	534.50	2.102
Max					0.088		0.739		2.571

## Data Availability

The original contributions presented in this study are included in the article. Further inquiries can be directed to the corresponding author.
